# Psychometric properties of the PERMA Profiler for measuring wellbeing in Australian adults

**DOI:** 10.1371/journal.pone.0225932

**Published:** 2019-12-23

**Authors:** Jillian Ryan, Rachel Curtis, Tim Olds, Sarah Edney, Corneel Vandelanotte, Ronald Plotnikoff, Carol Maher

**Affiliations:** 1 Precision Health Future Science Platform, Commonwealth Scientific and Industrial Research Organisation, Adelaide, South Australia, Australia; 2 Alliance for Research in Exercise, Nutrition, and Activity, University of South Australia, Adelaide, South Australia, Australia; 3 Physical Activity Research Group, School of Health Medical and Applied Sciences, Central Queensland University, Norman Gardens, Queensland, Australia; 4 Priority Research Centre for Physical Activity and Nutrition, The University of Newcastle, Callaghan, New South Wales, Australia; Monash University, AUSTRALIA

## Abstract

**Introduction:**

This study evaluated the psychometric properties of the PERMA Profiler, a 15-item self-report measurement tool designed to measure Seligman’s five pillars of wellbeing: Positive emotions, Relationships, Engagement, Meaning, and Accomplishment.

**Methods:**

Australian adults (N = 439) completed the PERMA Profiler and measures of physical and mental health (SF-12), depression, anxiety, stress (DASS 21), subjective physical activity (Active Australia Survey), and objective activity and sleep (GENEActiv accelerometer). Internal consistency was examined using Cronbach’s alpha and associations between theoretically related constructs examined using Pearson’s correlation. Model fit in comparison with theorised models was examined via Confirmatory Factor Analysis.

**Results:**

Results indicated acceptable internal consistency for overall PERMA Profiler scores and all subscales (α range = 0.80–0.93) except Engagement (α = 0.66). Moderate associations were found between PERMA Profiler wellbeing scores with subjective constructs (e.g. depression, anxiety, stress; *r* = -0.374 - -0.645, *p* = <0.001) but not objective physical activity or sleep. Data failed to meet model fit criteria for neither the theorised five-factor nor an alternative single-factor structure.

**Conclusions:**

Findings were mixed, providing strong support for the scale’s internal consistency and moderate support for congervent and divergent validity, albeit not in comparison to objectively captured activity outcomes. We could not replicate the theorised data structure nor an alternative, single factor structure. Results indicate insufficient psychometric properties of the PERMA Profiler.

## Introduction

With steadily increasing human life expectancies [1] has come growing interest in understanding how individuals can live better (happier, more satisfying) lives [2]. Wellbeing is an intangible, non-economic measure of success that captures not only the absence of disease, but the extent to which an individual is flourishing; engaged in life, has good relationships with others, experiences positive and negative emotions, and is resilient to adversity [3, 4]. A growing focus on wellbeing is now evident across scientific, policy, and commercial domains, for example, it is studied regularly in scientific research [5, 6], has informed the development of new psychotherapeutic treatments for depression [7], and has been used as a foundation for work- and community-based health promotion programs [8].

Although wellbeing is regularly studied in scientific research, there remain some challenges associated with how it is operationalised and measured at a population level [9], since it is entirely subjective and hasn’t yet been clearly defined. Wellbeing assessment tools are mostly limited to self-report surveys, which are associated with social report biases and other limitations. Nonetheless, a number of measurement instruments have been developed, the most recent of which is the PERMA Profiler [10], which captures Seligman’s five pillars of wellbeing: Positive emotion (experience of positive emotions), Engagement (being immersed in life pursuits), Relationships (having satisfying relationships with others), Meaning (working towards a bigger goal), and Accomplishment (regularly achieving successes) [11, 12]. PERMA is a highly regarded wellbeing model and therefore the PERMA Profiler has experienced fast uptake since it was published in 2016 [13, 14].

The bulk of available data regarding the PERMA Profiler’s psychometric properties is located in the original development and validation study [10]. In this study, eight samples of respondents (*n* range 166–23,692) completed the PERMA Profiler and responses were analysed to evaluate the scale’s internal and test-retest reliability, factor structure, and construct validity. Results established positive evidence of the scale’s internal consistency (Cronbach’s alpha of α = 0.94 averaged across pilot studies, *n* = 31,966) and test-retest stability (mean Pearson’s *r* of 0.78, *n* = 3,257). Acceptable construct validity was also demonstrated, with overall wellbeing scores correlating in the expected directions with theoretically related constructs, specifically, an alternative wellbeing scale (Flourishing Scale [14], *r* = 0.84) and measures of mental well-being (the Short Warwick-Edinburgh Mental Well-being Scale [15], *r* = 0.80). Factor analysis also confirmed adequate model fit for the five-factor structure (e.g. RMSEA = .055, TLI = .968; [10]).

Since then, other studies have set out to validate the PERMA Profiler for use with different populations including South Australian [16], Italian [17], Malaysian [18], and Turkish [19] respondents. Although these studies have generally found evidence of acceptable psychometric properties there are some gaps, including mixed findings regarding whether response data reliably loads onto the five PERMA factors. Of studies that investigated factor structure, one [17] but not another [18], was able to replicate the theorised five-factor structure via confirmatory factor analysis. In addition, no studies have tested the PERMA Profiler’s convergent validity against objective criterion (e.g. physical activity and sleep), despite the fact that wellbeing is theorised to positively correlate with physical activity levels and sleep duration [20]. There is therefore a need for further investigation into the psychometric properties of the PERMA Profiler.

The current study aims address some of the outstanding questions and contribute to the existing evidence regarding the PERMA Profiler’s psychometric properties. It was hypothesised that: **1)** Items will show internal consistency. **2)** The 15 core items will load onto the five sub-scales of Positive emotion, Engagement, Relationships, Meaning, and Accomplishment. **3)** PERMA Profiler scores will show convergent validity with subjective measures of health and mental-health related quality of life, physical activity, and sleep. **4)** Well-being scores will show divergent validity with subjective measures of depression, anxiety, and stress.

## Methodology

Ethical approval for the study was obtained from the Human Research Ethics Committee of the University of South Australia and the trial is registered with the Australian and New Zealand Clinical Trial Registry, protocol number: ACTRN12617000113358. All participants provided informed consent prior to participating in this research by selecting a box on an online form that indicated their understanding of the nature of the research and that they agreed to participate.

Data were collected during the baseline assessment of a large-scale Randomised Controlled Trial (RCT) that is currently evaluating a smartphone-delivered social and gamified physical activity intervention entitled “Active Team” (see study protocol, [21]). Briefly, participants (N = 444) were recruited to the RCT in clusters of 3–8 individuals based on existing relationships. Participants were primarily recruited via Facebook recruitment campaigns and word-of-mouth strategies (i.e., recruits invited others to join their teams). To meet eligibility criteria participants were required to be between 18 and 65 years old, Australian residents, self-reportedly inactive (<150 minutes of moderate-to-vigorous activity per week), and regular Facebook users. No other exclusion criteria applied.

Participants completed assessments of subjective outcomes via an online survey hosted by survey software, Qualtrics. After expressing interest in participating in the study, participants received a link to the survey via email. The first page of the survey provided an overview of the Active Team study and displayed the participant information sheet. After giving consent, participants could begin to fill out the survey, which was distributed over eight pages and assessed participant wellbeing (overall PERMA wellbeing score and five subscale scores), subjective physical activity, and experience of depression, stress, and anxiety.

**Wellbeing** was measured using the PERMA Profiler, a 15-item scale designed to measure Seligman’s five pillars of wellbeing including Positive and negative emotions, Engagement, Relationships, Meaning, and Accomplishment (three items per subscale) [10]. The PERMA Profiler contains an additional eight ‘filler’ items that capture negative emotion, loneliness, and overall happiness, but these are not reported in the current study. Response options for each question are measured on 11-item Likert scales that include single-word anchors at each end, for example, question A1 (‘*In general*, *how often do you feel joyful*?’) is anchored by ‘never’ & ‘always’. All items are worded in a positive direction whereby higher scores equate to better wellbeing. During data analysis an overall wellbeing score is calculated by creating an average score on each item and subscale scores are calculated by averaging the three items on each subscale.

**Depression, anxiety, and stress** was measured by the Depression Anxiety Stress Scale (DASS-21 [22]). The DASS-21 has 21 items and has also been shown to be reliable, for example, test-retest reliabiltiy coefficients ranging between 0.77–0.89 [23] and valid, for example, correlations between DASS-21 scores and convergent constructs r = 0.79 with Beck Depression Invetory [24]. Responses to items on each of the DASS 21 subscales were averaged to produce separate scores for Depression, Anxiety, and Stress. Scores can range from 0–56 with a higher score representing stronger experience of symptoms. **Quality of life** was captured using the Short Form 12-item Health Survey or SF-12 [25], which has shown to have adequate reliability including test-retest coefficients of 0.89 (physical component) and 0.76 (mental component) and validity including convergent validity with the Physical (*r* = 0.67), Psychological (*r* = 0.70), and Global (*r* = 0.78) components of the Sickness Impact Profile. Scores for mental and physical health were calculated according to standardised scoring instructions and adjusted for age. **Subjective physical activity** was assessed using the Active Australia Survey (AAS [26]). This 8-item scale captures respondents’ physical activity frequency, duration, intensity, and has shown to have adequate reliability including test-retest coefficients of 0.52 (95% CI, 0.44–0.60 [27]) and convergent validity with accelerometer-measured physical activity (*r =* 0.49–0.64, *p* < 0.01 [28]).

**Objective measures of physical activity and sleep** were taken using GENEActiv accelerometers [Activinsights Ltd., UK], which were administered using a hands-off, standardised protocol in which research staff communicated with participants via phone and email and used express post to send and receive accelerometers. Participants were instructed to wear the GENEActiv accelerometer on their non-dominant wrist for seven consecutive days whilst completing an activity diary to manually record their sleep, wake, and nap times. Upon return of each accelerometer data were downloaded and screened for compliance to minimum wear times (10 hours over at least four days including at least one weekend-day). Participants who returned insufficient data were asked to wear the accelerometer for a second or third time and excluded from the study if they failed to return sufficient data after three attempts. Data were processed using custom Matlab software and according to established cutpoints which calculated minutes spent in light, moderate, and vigorous physical activity, and sleep [29], weighted such that mean scores took into account the imbalance of weekdays (5) and weekend days (2). For participants who did not wear the accelerometer overnight, sleep onset and wake times were manually adjusted based on their activity diaries. For the purposes of the current study, moderate and vigorous physical activity were summed to produce an estimate of Moderate-Vigorous Physical Activity (MVPA) and minutes spent in sleep were included as a separate outcome.

Five participants did not complete the PERMA Profiler, therefore analyses include 439 participants.

## Data analysis

Analyses were completed in SPSS (version 25), except for confirmatory and exploratory factor analyses, which were completed in MPlus 7.3. Participants’ outcome data were analysed descriptively and compared to population norms to determine whether scores differed significantly to comparable samples’ scores on wellbeing [10], depression, anxiety, and stress [30], quality of life [31], subjective physical activity [32], objective physical activity [33], and sleep duration [34].

### Internal consistency

Cronbach’s alpha was calculated to indicate internal consistency as a measure of reliability for each of the sub-scales and overall well-being.

### Factor structure

Sample adequacy tests (Bartlett’s test of sphericity and Kaiser-Meyer-Olkin measure of sampling adequacy) were carried out to determine appropriateness of factor analysis with the given data characteristics. Factor structure was examined using confirmatory factor analysis with maximum likelihood estimation. The five 3-item PERMA factors were specified with each item loading only on its respective factor. The latent factors were specified to covary. Chi-square is sensitive to large sample sizes, therefore model fit was considered acceptable if Comparative Fit Index (CFI) >.95, Root Mean Squared Error of Approximation (RMSEA) < .06, and Standardized Root Mean Square Residual (SRMR) < .08 [35].

Since the data did not fit the hypothesized model, supplementary analyses were undertaken to further elucidate the factor structure of the PERMA Profiler. Confirmatory factor analysis examined whether the 15 core items loaded onto a single well-being factor, using maximum likelihood estimation and the fit indices described above. Pearson’s correlations examined inter-item associations. Since the data did not fit this model either, additional exploratory factor analysis was conducted using maximum likelihood estimation and geomin oblique rotation (latent factors were expected to correlate and include potential item cross-loadings). The number of factors was determined by examining a scree plot of eigenvalues and parallel analysis [36].

### Convergent and divergent construct validity

Construct validity was assessed by examining Pearson’s correlations between PERMA Profiler well-being scores and other theoretically relevant outcomes, where *r* = .00 - .30 was considered a negligible correlation, *r* = .30 - .50 considered a weak association, *r* = .50 - .70 considered a moderate correlation and *r* = >.70 considered a strong correlation [37]. Convergent constructs included self-reported physical and mental health (measured by SF-12) and objectively measured sleep and physical activity whilst divergent outcomes included depression, anxiety, and stress symptomology (measured by DASS-21).

## Results

### Participants

The majority of participants were female (74.0%), had a university degree (53.8%), and were overweight or obese (76.4%). Participants’ mean age and standard deviation (SD) was 41.3 (SD 11.6). Participants’ physical activity and sleep levels, as well as scores on the PERMA Profiler, SF-12, and DASS 21 are shown in Table 1. Compared to population norms [10], participants scored significantly lower on each of the PERMA Profiler subscales and for overall wellbeing (e.g. mean overall well-being score of 6.6, SD 1.5 compared to population norm M 7.0, SD 1.6). Consistent with this, participants scored significantly higher on depression, anxiety, and stress than other populations, although mean scores on each of these were within the ‘normal’ range [22]. Accelerometer data indicated that participants completed a mean of 105.7 (SD 50.6) minutes of MVPA and slept for about 531 minutes (SD 56; about 8 hours, 51 minutes) per day. Quality of life, subjectively- and objectively- measured physical activity scores did not differ significantly from comparable populations (see Table 1).

**Table 1:** Mean scores on PERMA Profiler and divergent and convergent constructs (N = 439).

Constructs		Sample mean scores (SD)	Population norm mean scores (SD)
Wellbeing (PERMA Profiler)			
	Overall well-being	6.6 (1.5)	7.0 (1.6)^a^**[10]
	Positive emotion	6.4 (1.7)	6.7 (2.0)^a^**
	Engagement	6.5 (1.6)	7.3 (1.7)^a^**
	Relationships	7.1 (1.9)	6.9 (2.1)^a^
	Meaning	6.8 (1.9)	7.1 (2.1)^a^**
	Accomplishment	6.3 (1.6)	7.3 (1.7)^a^**
Depression, Anxiety, and Stress (DASS-21)			
	Depression	8.0 (7.7)	5.1 (7.7)^b^**[30]
	Anxiety	6.0 (6.0)	3.5 (5.6)^b^**
	Stress	12.5 (8.0)	8.0 (8.5)^b^**
Quality of life (SF-12)			
	Mental health	47.6 (8.8)	52.4 (8.8)^c^ [31]
	Physical health	46.2 (8.4)	48.9 (10.2)^c^
Subjective MVPA minutes/day (AAS)		37.2 (35.6);	38.7 (88.51)^d^ [32]
Objective MVPA minutes/day (accelerometer-measured)		105.7 (50.6)	105.9 (51.3)^e^ [33]
Sleep duration minutes/day (accelerometer-measured)		531.3 (95.0)	501 (56)^f^** [34]

**p = <0.001

a. Compared to n = 2593 Australia adults

b. Compared to n = 497 Australian adults

c. Compared to n = 2013 South Australian adults

d. Compared to 19,259 Australian adults

e. Compared to n = 23,192 non-clinical male and female British adults (pooled male and female mean/variance is reported)

f. Compared to n = 1358 Australian adults.

### Internal consistency

Cronbach’s alphas showed moderate to strong internal consistency for Positive emotions (3 items, α = .87), Relationships (3 items, α = .82), Meaning (3 items, α = .80), Accomplishment (3 items, α = .72) and overall Well-being (15 items, α = .93) subscales. On the other hand, internal consistency for the engagement subscale was weak (3 items, α = .66).

### Factor structure

Bartlett’s test of sphericity was significant (*χ*2 (105) = 4662.631, *p*<0.001), indicating that it was appropriate to use the factor analytic model on this set of data. The Kaiser-Meyer-Olkin measure of sampling adequacy indicated that the strength of the relationships among variables was high (KMO = .934), thus it was acceptable to proceed with the analysis.

The solution for the confirmatory factor analysis examining the five-factor structure of the PERMA was not admissible due to a non-positive definite psi matrix. This indicates that the model is miss-specified and does not fit the data. The results are not interpretable and therefore not reported. Examination of the output did not reveal a negative residual variance or correlation greater than 1, therefore there was likely a linear dependency between more than two latent factors. Fewer factors were likely required.

Given the PERMA Profiler is also suggested to measure overall well-being, confirmatory factor analysis examined whether the 15 items loaded onto a single well-being factor. The model did not fit the data well (χ^2^ (90) 903.22, *p* < .001, CFI .82, RMSEA .14, SRMR .07). Standardised factor loadings ranged from .41 to .85.

Examination of bivariate correlations between the items (S1 Table) did not reveal an obvious clustering of items; some items showed high correlations with items from multiple proposed PERMA factors. Exploratory factor analysis was therefore conducted to identify the most appropriate number of factors from the data. The scree plot and parallel analysis (S2 Table) suggested that two factors should be retained. The 2-factor solution provided a moderate fit to the data with CFI near acceptable values and an acceptable SRMR (χ^2^(76) 444.50, *p* < .001, CFI .92, RMSEA .11, SRMR .04). Rotated factor loadings are shown in Table 2. Factor 1 consisted of all three items on the Engagement, Meaning, and Accomplishment subscales and two items from the Purpose subscale, while Factor 2 consisted of one item from the Purpose subscale and two items from the Relationship subscale.

**Table 2:** Correlation matrix displaying relationships between PERMA-Profiler scores and convergent and divergent constructs.

	1	2	3	4	5	6	7	8	9	10	11	12	13	14
1. PERMA: Overall well-being	1.00													
2. PERMA: Positive emotions	0.921**	1.00												
3. PERMA: Engagement	0.791**	0.676**	1.00											
4. PERMA: Relationships	0.809**	0.744**	0.463**	1.00										
5. PERMA: Meaning	0.914**	0.813**	0.637**	0.718**	1.00									
6. PERMA: Accomplishments	0.774**	0.630**	0.609**	0.421**	0.661**	1.00								
7. DASS-21: Depression	-0.645**	-0.660**	-0.444**	-0.493**	-0.615**	-0.493**	1.00							
8. DASS-21: Anxiety	-0.374**	-0.383**	-0.252**	-0.291**	-0.346**	-0.291**	0.656**	1.00						
9. DASS-21: Stress	-0.460**	-0.275**	-0.275**	-0.349**	-0.416**	-0.349**	0.720**	0.683**	1.00					
10. SF-12: Physical health	0.152**	0.122*	0.095*	0.075	0.152	0.210**	-0.212**	-0.278**	-0.185**	1.00				
11. SF-12: Mental health	0.633**	0.678**	0.457**	0.484**	0.554**	0.484**	-0.698**	-0.470**	0.613**	-0.034	1.00			
12. AAS: Subjective MVPA	0.117*	0.113*	0.101*	0.067*	0.115*	0.098*	-0.045	0.057	0.011	0.120*	0.092	1.00		
13. Objective MVPA	0.026	0.045	-0.007	0.067	0.012	-0.021	-0.026	0.004	0.012	0.107*	0.015	0.103*	1.00	
14. Objective Sleep	-0.047	-0.046	-0.029	-0.058	-0.064	0.007	0.057	0.033	-0.005	-0.040	-0.106*	0.015	-0.170**	1.00

*p <0.05

**p <0.001

The two factors were highly correlated (r = 0.62) and eight items showed statistically significant cross-loadings. According to the .40-.30-.20 rule of thumb (Howard 2016), two of these items showed unsatisfactory loadings (P3 showed a cross-loading greater than .30 and R1 showed a difference in loading between the primary and alternative factor of less than .20).

### Convergent and divergent construct validity

PERMA Profiler scores showed moderate and statistically significant convergent validity with scores on self-reported measures of physical and mental health (r = 0.46 to 0.68; see Table 2). The strongest associations were seen between PERMA wellbeing scores with depression scores (DASS-21; r = -0.645, p = <0.001) and mental health scores (SF-12 MCS, r = 0.633, p = <0.001). Negligible correlations, on the other hand, were observed between PERMA wellbeing scores with either of the objectively measured outcomes, physical activity (r = -0.026) and sleep (r = -0.047).

## Discussion

This study examined the reliability and validity of the PERMA Profiler as a measure of well-being in 439 Australian adults. Findings were mixed, and although results generally supported the scale’s internal consistency and convergent and divergent validity with regards to subjectively measured constructs, they did not support hypothesised relationships between PERMA Profiler scores and objective measures of sleep or physical activity and data did not support either of two hypothesised data structures. A discussion of the implications of these findings and potential explanations will follow.

Our results suggest that the PERMA Profiler has good internal consistency for overall scores and all subscale scores with the exception of engagement and this pattern is consistent with existing evidence [10, 16, 17]. Limited internal consistency can indicate problematic item wording or item sampling, for example, respondents may be misinterpreting items and responding differently to different items on the same scale, or the questions may not sample the construct of engagement well. Further research using differently worded versions of the engagement items is needed to test and potentially improve the reliability of the Engagement subscale. In the meantime, researchers should be cautious about interpreting engagement scores independently.

Contrary to expectations, our data did not fit the theorised data structure regardless if a five- or one- factor structure was tested. Examination of the bivariate item correlations highlight the Accomplishment and Engagement subscales as potential sources of discordance, with each of these factors containing two and three item-correlations lower than r = .50, respectively. Possible explanations include that within the Engagement subscale, item E2 is double-barrelled (In general, to what extent do you feel excited and interested in things?) while E1 (How often do you become absorbed in what you are doing?) and E3 (How often do you lose track of time while doing something you enjoy) refer to a single affective state (task absorption). It is further possible that E2 may be capturing respondents’ transient levels of arousal (‘excited and interested’) rather than a stable level of occupation with- or attention to- task, which is how the construct is operationalised (10). The Accomplishment subscale contains similarly low item correlations, although A1 (How much of the time do you feel you are making progress towards accomplishing your goals?) and A2 (How often do you achieve the important goals you have set for yourself?) are more highly correlated (r = .67). A possible explanation is that both A1 and A2 refer to self-determined goals whilst A3 (How often are you able to handle your responsibilities?) refers to responsibilities that may be perceived as obligations rather than ‘accomplishments’.

One alternative model that contained two factors was derived from exploratory factor analysis, however, this model contained just two primary loadings, suggesting that this is not a good alternative solution either. Examination of item loadings in this two-factor model suggest that one factor potentially relates to the appraisal of activities, ambitions, and goals (e.g. a broad ‘purpose’ factor), whilst the second factor relates more to respondent satisfaction with relationships and personal life (e.g. a broad ‘relationships’ factor, Table 3). Few existing studies have tested PERMA Profiler data against the theorised structure and findings have been mixed. While data in the original development and validation study, as well as a subsequent and independent validation study [17] did satisfy 5-factor model fit criteria, data in one other study did not satisfy model fit for the theorised models and instead supported a three-factor model that consisted of Positive emotions/Relationships, Meaning/Accomplishment, and Engagement [18]. Participants in the one study that has replicated the five-factor model structure (17) were university students in Italy, with a lower mean age (26.4, SD 3.0) and narrower range (19–40) than the current sample. Differential bias could be at play here, whereby the reliability of the factor structure differs depending on the age of participants. Further investigation is warranted.

**Table 3:** Geomin rotated factor loadings (standard errors) and item residuals for the 2-factor solution.

Item	Factor 1		Factor 2		Residuals
P1	0.70*	(0.04)	0.13*	(0.05)	0.38
P2	0.78*	(0.04)	0.07	(0.05)	0.32
P3	0.46*	(0.04)	0.53*	(0.04)	0.20
E1	0.69*	(0.06)	-0.25*	(0.07)	0.68
E2	0.85*	(0.02)	-0.00	(0.01)	0.27
E3	0.48*	(0.06)	-0.09	(0.06)	0.81
R1	0.41*	(0.05)	0.29*	(0.06)	0.59
R2	0.17*	(0.05)	0.73*	(0.04)	0.29
R3	0.00	(0.00)	0.93*	(0.02)	0.13
M1	0.79*	(0.04)	0.08	(0.05)	0.29
M2	0.78*	(0.04)	0.08	(0.05)	0.29
M3	0.72*	(0.04)	0.18*	(0.05)	0.29
A1	0.82*	(0.05)	-0.20*	(0.06)	0.49
A2	0.77*	(0.05)	-0.20*	(0.07)	0.55
A3	0.53*	(0.06)	-0.09	(0.06)	0.77

*p < .05

Unexpected results such as data not following the theorised structure can sometimes be attributable to peculiarities of the sample. As shown in Table 1, our sample differed from population norms in a number of ways, including that participants reported significantly lower PERMA wellbeing and subscale scores and significantly higher depression, anxiety, and stress scores, although these were still within the normal range. Whilst these differences serve as a potential explanation for the unclear data structure, they are unlikely to explain the finding fully as none of the characteristics exclude our sample from being eligible to complete the PERMA Profiler [10]. Some of the wording and anchors for PERMA Profiler items were amended after validation, for example, the prefix ‘In general,’ was added to some items and the wording of R2 was changed from “To what extent have you been feeling loved?” to “To what extent do you feel loved?” Although these are relatively minor amendments, it is possible that they may have introduced some unanticipated measurement effects. Further independent research that uses the final version of the PERMA Profiler is needed to validate its factor structure with different samples. In addition, item analysis that assesses differential item functioning would be valuable to elucidate understanding of the psychometric properties of individual items.

Although our data did not support the PERMA Profiler’s factor structure, findings generally supported convergent and divergent construct validity, with scores associating in the expected directions with all subjective (but not objective) constructs tested. Consistent with previous research [17], overall wellbeing scores were most strongly associated with depression and stress and general mental health. Contrary to expectations, however, few associations were found between PERMA Profiler wellbeing scores and objectively measured sleep and physical activity. Again, there are a number of potential explanations for this unexpected result. Although an association between wellbeing and sleep and physical activity is theorised in the literature, activity and sleep are often measured subjectively, and consistent with this, we found that subjectively measured physical activity was significantly associated with PERMA Profiler wellbeing scores even though objectively measured activity was not. When measured objectively, other studies have generally reported a weak or negligible relationship between activity and sleep with wellbeing as measured by the Satisfaction with Life Scale [38, 39]. MVPA estimates may suggest our sample was quite active at baseline, however this is likely an artefact of using GENEActiv accelerometers and Esliger et al cut-points ([29]; which are validated and widely used with GeneActiv accelerometry data). Estimates of MVPA can vary by an order of magnitude according to which accelerometers, validated cut-points, epoch lengths and processing algorithms are used. Although estimates of mean values can vary a lot, they are typically highly correlated, and therefore have little effect on correlations with outcomes. The MVPA estimates in the current study are actually lower than Australian adult population norms collected using GENEActiv accelerometers using Esliger’s cut offs [40]. These findings point to a need for further evidence regarding the presence or absence of associations between objectively measured activity and subjective wellbeing.

### Strengths & limitations

Strengths of this study include that we had a large sample who completed all assessments, including for the first time in a study that uses PERMA Profiler, objectively measured physical activity and sleep. There are also some limitations that should be acknowledged. The sample was biased towards well-educated females and there were a number of statistically significant differences between our sample and population norms including lower PERMA wellbeing and subscale scores, higher depression, anxiety, and stress scores, and greater mean sleep duration. Within our sample, however, PERMA scores did not vary significantly depending on gender (male or female), age (based on mean split), or education category.

### Conclusions

Measuring wellbeing at the population level is an important research priority. Although the recently developed PERMA Profiler appears to be a strong tool due to its foundation in Seligman’s PERMA wellbeing model, researchers should be aware of the inconsistencies in the scale’s psychometric property reported in this study and others. Taken in context with findings from previous studies our results call into question the five-factor structure of the PERMA Profiler in its current format, particularly given mixed evidence regarding whether PERMA Profiler response data loads onto the theoretical model. Consistent correlations with convergent and divergent constructs in the theorised direction suggest promise for clinical and theoretical utility, particularly given the minimal burden associated with completing this measure in its short form. These results highlight a need for further independent validation studies, particularly those that delve deeper into the factor structure of the PERMA Profiler.

## Supporting information

S1 Table PERMA-Profiler item correlations. (DOCX)

S2 Table Actual and random eigenvalues from parallel analysis. (DOCX)

## References

[1] Oeppen J, Vaupel JW. Broken limits to life expectancy. 2002;296(5570):2 10.1126/science.1069675 12004104

[2] Pinto S, Fumincelli L, Mazzo A, Caldeira S, Martins JC. Comfort, well-being and quality of life: Discussion of the differences and similarities among the concepts. Porto Biomedical Journal. 2017;2(1):6–12. 10.1016/j.pbj.2016.11.003 PMC6806988 32258577

[3] Diener E, Seligman ME. Beyond money: Toward an economy of well-being. Psychological science in the public interest. 2004;5(1):1–31. 10.1111/j.0963-7214.2004.00501001.x 26158992

[4] Huppert FA. The state of wellbeing science: Concepts, measures, interventions, and policies. Wellbeing: A complete reference guide. 2014:1–49.

[5] Boehm JK, Kubzansky LD. The heart's content: the association between positive psychological well-being and cardiovascular health. Psychological bulletin. 2012;138(4):655 10.1037/a0027448 22506752

[6] Windle G, Hughes D, Linck P, Russell I, Woods B. Is exercise effective in promoting mental well-being in older age? A systematic review. Aging & mental health. 2010;14(6):652–69. 20686977 10.1080/13607861003713232

[7] Zilcha-Mano S, Dinger U, McCarthy KS, Barrett MS, Barber JP. Changes in well-being and quality of life in a randomized trial comparing dynamic psychotherapy and pharmacotherapy for major depressive disorder. Journal of affective disorders. 2014;152:538–42. 10.1016/j.jad.2013.10.015 24176534 PMC4423535

[8] Carolan S, Harris PR, Cavanagh K. Improving employee well-being and effectiveness: systematic review and meta-analysis of web-based psychological interventions delivered in the workplace. Journal of medical Internet research. 2017;19(7):e271 10.2196/jmir.7583 28747293 PMC5550734

[9] Dodge R, Daly AP, Huyton J, Sanders LD. The challenge of defining wellbeing. International journal of wellbeing. 2012;2(3).

[10] Butler J, Kern ML. The PERMA-Profiler: A brief multidimensional measure of flourishing. International Journal of Wellbeing. 2016;6(3).

[11] Seligman ME. Positive psychology, positive prevention, and positive therapy. Handbook of positive psychology. 2002;2(2002):3–12.

[12] Seligman ME. Flourish: A visionary new understanding of happiness and well-being: Simon and Schuster; 2012.

[13] Lai MK, Leung C, Kwok SY, Hui AN, Lo HH, Leung JT, et al A multidimensional PERMA-H positive education model, general satisfaction of school life, and character strengths use in Hong Kong senior primary school students: Confirmatory factor analysis and path analysis using the APASO-II. Frontiers in psychology. 2018;9:1090 10.3389/fpsyg.2018.01090 30008690 PMC6034423

[14] Sun J, Kaufman SB, Smillie LD. Unique associations between big five personality aspects and multiple dimensions of well‐being. Journal of personality. 2018;86(2):158–72. 10.1111/jopy.12301 28035671

[15] Stewart-Brown S, Tennant A, Tennant R, Platt S, Parkinson J, Weich S. Internal construct validity of the Warwick-Edinburgh mental well-being scale (WEMWBS): a Rasch analysis using data from the Scottish health education population survey. Health and quality of life outcomes. 2009;7(1):15. 19228398 10.1186/1477-7525-7-15 PMC2669062

[16] Iasiello M, Bartholomaeus J, Jarden A, Kelly G. Measuring PERMA+ in South Australia, the State of Wellbeing: A comparison with national and international norms. Journal of Positive Psychology and Wellbeing. 2017;1(2):| 53–72.

[17] Giangrasso B. Psychometric properties of the PERMA-Profiler as hedonic and eudaimonic well-being measure in an Italian context. Current Psychology. 2018:1–10.

[18] Khaw D, Kern M. A cross-cultural comparison of the PERMA model of well-being. Undergraduate Journal of Psychology at Berkeley, University of California. 2014;8:10–23.

[19] Ayse EB. Adaptation of the PERMA Well-Being Scale into Turkish: Validity and Reliability Studies. Educational Research and Reviews. 2018;13(4):129–35.

[20] Haapasalo V, de Vries H, Vandelanotte C, Rosenkranz RR, Duncan MJ. Cross-sectional associations between multiple lifestyle behaviours and excellent well-being in Australian adults. Preventive medicine. 2018;116:119–25. 10.1016/j.ypmed.2018.09.003 30218725

[21] Edney S, Plotnikoff R, Vandelanotte C, Olds T, De Bourdeaudhuij I, Ryan J, et al “Active Team” a social and gamified app-based physical activity intervention: randomised controlled trial study protocol. BMC public health. 2017;17(1):859 10.1186/s12889-017-4882-7 29096614 PMC5667488

[22] Lovibond PF, Lovibond SH. The structure of negative emotional states: Comparison of the Depression Anxiety Stress Scales (DASS) with the Beck Depression and Anxiety Inventories. Behaviour research and therapy. 1995;33(3):335–43. 10.1016/0005-7967(94)00075-u 7726811

[23] Asghari A, Saed F, Dibajnia P. Psychometric properties of the Depression Anxiety Stress Scales-21 (DASS-21) in a non-clinical Iranian sample. Int J Psychol. 2008;2(2):82–102.

[24] Antony MM, Bieling PJ, Cox BJ, Enns MW, Swinson RP. Psychometric properties of the 42-item and 21-item versions of the Depression Anxiety Stress Scales in clinical groups and a community sample. Psychological assessment. 1998;10(2):176.

[25] Ware JE Jr, Kosinski M, Keller SD. A 12-Item Short-Form Health Survey: construction of scales and preliminary tests of reliability and validity. Medical care. 1996:220–33. 10.1097/00005650-199603000-00003 8628042

[26] Australian Institute of Health and Welfare. The Active Australia Survey: a guide and manual for implementation, analysis and reporting. Canberra: Australian Government, 2003.

[27] Brown W, Trost S, Bauman A, Mummery K, Owen N. Test-retest reliability of four physical activity measures used in population surveys. Journal of Science and Medicine in Sport. 2004;7(2):205–15. 10.1016/s1440-2440(04)80010-0 15362316

[28] Freene N, Waddington G, Chesworth W, Davey R, Cochrane T. Validating two self-report physical activity measures in middle-aged adults completing a group exercise or home-based physical activity program. Journal of science and medicine in sport. 2014;17(6):611–6. 10.1016/j.jsams.2013.11.002 24332192

[29] Esliger DW, Rowlands AV, Hurst TL, Catt M, Murray P, Eston RG. Validation of the GENEA Accelerometer. 2011. 10.1249/MSS.0b013e31820513be 21088628

[30] Crawford J, Cayley C, Lovibond PF, Wilson PH, Hartley C. Percentile norms and accompanying interval estimates from an Australian general adult population sample for self‐report mood scales (BAI, BDI, CRSD, CES‐D, DASS, DASS‐21, STAI‐X, STAI‐Y, SRDS, and SRAS). Australian Psychologist. 2011;46(1):3–14.

[31] Avery J, Dal Grande E, Taylor A. Quality Of Life in South Australia as Measured by the SF12 Health Status Questionnaire. 2004.

[32] Australian Bureau of Statistics. Physical Activity. 2018.

[33] Cassidy S, Fuller H, Chau J, Catt M, Bauman A, Trenell MI. Accelerometer-derived physical activity in those with cardio-metabolic disease compared to healthy adults: a UK Biobank study of 52,556 participants. Acta diabetologica. 2018;55(9):975–9. 10.1007/s00592-018-1161-8 29808390 PMC6096713

[34] Matricciani L, Fraysse F, Grobler AC, Muller J, Wake M, Olds T. Sleep: population epidemiology and concordance in Australian children aged 11–12 years and their parents. BMJ open. 2019;9(Suppl 3):127–35. 10.1136/bmjopen-2017-020895 31273023 PMC6624061

[35] Lt Hu, Bentler PM. Cutoff criteria for fit indexes in covariance structure analysis: Conventional criteria versus new alternatives. Structural equation modeling: a multidisciplinary journal. 1999;6(1):1–55.

[36] Howard MC. A review of exploratory factor analysis decisions and overview of current practices: What we are doing and how can we improve? International Journal of Human-Computer Interaction. 2016;32(1):51–62.

[37] Mukaka MM. A guide to appropriate use of correlation coefficient in medical research. Malawi Medical Journal. 2012;24(3):69–71. 23638278 PMC3576830

[38] Lemola S, Ledermann T, Friedman EM. Variability of sleep duration is related to subjective sleep quality and subjective well-being: an actigraphy study. PloS one. 2013;8(8):e71292 10.1371/journal.pone.0071292 23967186 PMC3743871

[39] Withall J, Stathi A, Davis M, Coulson J, Thompson J, Fox K. Objective indicators of physical activity and sedentary time and associations with subjective well-being in adults aged 70 and over. International journal of environmental research and public health. 2014;11(1):643–56. 10.3390/ijerph110100643 24452258 PMC3924465

[40] Fraysse F, Grobler AC, Muller J, Wake M, Olds T. Physical activity and sedentary activity: population epidemiology and concordance in Australian children aged 11–12 years and their parents. BMJ open. 2019;9(Suppl 3):136–46. 10.1136/bmjopen-2018-023194 31273024 PMC6624037

