# Supplementary material for: Psychometric properties of the PERMA Profiler for measuring wellbeing in Australian adults
Source: PLoS One. 2019 Dec 23;14(12):e0225932. doi: 10.1371/journal.pone.0225932 (PMC6927648; doi:10.1371/journal.pone.0225932)
Supplement: S1 Table — (DOCX) [file pone.0225932.s001.docx]

*Supplementary Table 1*. PERMA-Profiler item correlations

| Item | P1 | P2 | P3 | E1 | E2 | E3 | R1 | R2 | R3 | M1 | M2 | M3 | A1 | A2 | A3 |
| --- | --- | --- | --- | --- | --- | --- | --- | --- | --- | --- | --- | --- | --- | --- | --- |
| P1 | 1.00 |  |  |  |  |  |  |  |  |  |  |  |  |  |  |
| P2 | .72 | 1.00 |  |  |  |  |  |  |  |  |  |  |  |  |  |
| P3 | .68 | .69 | 1.00 |  |  |  |  |  |  |  |  |  |  |  |  |
| E1 | .40 | .39 | .36 | 1.00 |  |  |  |  |  |  |  |  |  |  |  |
| E2 | .73 | .73 | .69 | .43 | 1.00 |  |  |  |  |  |  |  |  |  |  |
| E3 | .41 | .34 | .33 | .38 | .41 | 1.00 |  |  |  |  |  |  |  |  |  |
| R1 | .49 | .47 | .55 | .24 | .54 | .26 | 1.00 |  |  |  |  |  |  |  |  |
| R2 | .53 | .54 | .69 | .23 | .51 | .21 | .54 | 1.00 |  |  |  |  |  |  |  |
| R3 | .54 | .52 | .77 | .19 | .49 | .21 | .50 | .79 | 1.00 |  |  |  |  |  |  |
| M1 | .63 | .68 | .69 | .38 | .70 | .28 | .55 | .57 | .52 | 1.00 |  |  |  |  |  |
| M2 | .61 | .67 | .67 | .41 | .73 | .29 | .64 | .59 | .52 | .79 | 1.00 |  |  |  |  |
| M3 | .60 | .66 | .74 | .39 | .70 | .35 | .50 | .61 | .58 | .74 | .74 | 1.00 |  |  |  |
| A1 | .53 | .55 | .47 | .53 | .54 | .31 | .36 | .36 | .31 | .56 | .52 | .56 | 1.00 |  |  |
| A2 | .47 | .49 | .42 | .51 | .52 | .29 | .25 | .34 | .30 | .54 | .48 | .53 | .67 | 1.00 |  |
| A3 | .31 | .49 | .33 | .27 | .39 | .16*^a^* | .20 | .26 | .24 | .39 | .39 | .38 | .37 | .33 | 1.00 |

Note: all *p*s <.001, except *^a^p* < .01
