# Supplementary material for: Psychometric properties of the PERMA Profiler for measuring wellbeing in Australian adults
Source: PLoS One. 2019 Dec 23;14(12):e0225932. doi: 10.1371/journal.pone.0225932 (PMC6927648; doi:10.1371/journal.pone.0225932)
Supplement: S2 Table — (DOCX) [file pone.0225932.s002.docx]

*Supplementary Table 2*. Actual and random eigenvalues from parallel analysis

| Factors | Eigenvalues | | |
| --- | --- | --- | --- |
|  | Actual | Average | 95^th^ percentile |
| 1 | 8.03 | 1.33 | 1.39 |
| 2 | 1.48 | 1.25 | 1.31 |
| 3 | 0.92 | 1.20 | 1.24 |
| 4 | 0.76 | 1.15 | 1.18 |
| 5 | 0.68 | 1.11 | 1.14 |
| 6 | 0.57 | 1.06 | 1.10 |
| 7 | 0.47 | 1.03 | 1.05 |
| 8 | 0.45 | 0.99 | 1.02 |
| 9 | 0.32 | 0.95 | 0.98 |
| 10 | 0.28 | 0.92 | 0.95 |
| 11 | 0.25 | 0.88 | 0.91 |
| 12 | 0.24 | 0.85 | 0.88 |
| 13 | 0.22 | 0.81 | 0.84 |
| 14 | 0.18 | 0.77 | 0.81 |
| 15 | 0.14 | 0.71 | 0.76 |
